# A New Phenylazo-Based Fluorescent Probe for Sensitive Detection of Hypochlorous Acid in Aqueous Solution

**DOI:** 10.3390/molecules27092978

**Published:** 2022-05-06

**Authors:** Qiuchen Liu, Chang Liu, Song He, Liancheng Zhao, Xianshun Zeng, Jin Zhou, Jin Gong

**Affiliations:** 1 School of Pharmacy, Weifang Medical University, Weifang 261053, China; qiuchen-liu@163.com; 2 Tianjin Key Laboratory for Photoelectric Materials and Devices, School of Materials Science & Engineering, Tianjin University of Technology, Tianjin 300384, China; kmn667@163.com (C.L.); hesong@tjut.edu.cn (S.H.); lczhao@hit.edu.cn (L.Z.); 3 School of Chemical Engineering and Technology, Tianjin University, Tianjin 300072, China; 4 School of Materials Science and Engineering, Harbin Institute of Technology, Harbin 150001, China

**Keywords:** hypochlorous acid, phenylazo, fluorescent probe, cell imaging, mitochondria

## Abstract

In this paper, we designed and synthesized a novel phenylazo-based fluorescent probe (RHN) for the sensing and imaging of hypochlorous acid (HClO) in mitochondria in living cells. In this process, HClO promoted the oxidation of the phenylazo group to generate a free Rhodol fluorophore moiety, which in turn restored strong fluorescence and realized the detection of HClO. As expected, RHN exhibited high selectivity, high sensitivity and rapid response, with detection limits as low as 22 nM (1.155 ng/mL). Importantly, the results of the cell imaging experiments indicated that RHN has the ability to image and sense HClO in mitochondria, which is of great significance for exploration of the specific role of HClO in both the immune system and diseases.

## 1. Introduction

As an important reactive oxygen species (ROS), hypochlorous acid (HClO) not only acts as a disinfectant and bleach in daily life, but also participates in various physiological processes (such as immune defenses and inflammatory responses) [1,2]. Although HClO plays a critical role in killing a variety of pathogens, abnormal levels of HClO could cause oxidative stress leading to a variety of human diseases, including tissue damage, kidney disease, atherosclerosis, cystic fibrosis, lung injury, rheumatoid arthritis and certain cancers [3,4]. Meanwhile, there is currently evidence that HClO is mainly present in mitochondria, because myeloperoxidase (MPO, which catalyzes the production of HClO from hydrogen peroxide and chloride ions) was found in the mitochondria of macrophages [5,6]. Therefore, the development of mitochondria-targeted fluorescent probes to monitor HClO in mitochondria is of great significance for exploring its production, its specific role in the immune system and the detection of diseases.

Although there were conventional techniques for quantifying HClO (e.g., potentiometric and electroanalytical), none of them were suitable for real-time and in situ detection of HClO in living cells. Compared with traditional HClO detection methods, the non-invasive fluorescence imaging techniques offer distinct advantages, such as ease of application both in solutions and living organisms, with high selectivity and sensitivity and temporal and spatial resolution for trace analytes [7,8,9]. Currently, based on the strong oxidizing properties of HClO, many fluorescent probes have been developed and applied to the sensing of HClO [10,11,12,13,14,15,16,17,18,19,20,21,22,23,24,25,26,27]. In fact, most of these reported probes are still limited. As shown in Table S1, some probes require the addition of large amounts of organic solvents as co-solvents, limiting their application in biological systems [10,12,13,15,18,19,20,21,25]. Some probes have poor detection limits and long response times, which affect the sensitivity and real-time performance of probe detection [13,14,15,18,19,24,25]. At the same time, most probes have low fluorescence enhancement multiples, which cannot effectively avoid the influence of background fluorescence, thus limiting their application in biological systems [11,12,13,14,15,17,18,19,24,25]. Therefore, the development of novel fluorescent probes, with high selectivity and high sensitivity for real-time detection of HClO in mitochondria, still has both great challenges and practical significance.

Recently, Sun et al. reported the first pilot work on the construction of a novel fluorescent probe based on the azo group, for detection of HClO with high selectivity and high sensitivity [28]. Inspired and encouraged by this work, we designed and synthesized a novel phenylazo-based fluorescent probe RHN for the sensing and imaging of HClO in mitochondria in living cells. In this process, HClO promoted the oxidation of the azo group to generate a free Rhodol fluorophore moiety, which in turn restored strong fluorescence and realizes the detection of HClO. As expected, RHN exhibited high selectivity, high sensitivity and rapid response, with detection limits as low as 22 nM (1.155 ng/mL). Importantly, the results of the cell imaging experiments indicated that RHN has the ability to image and sense HClO in mitochondria, which is of great significance for exploring the specific role of HClO in the immune system and diseases. 

## 2. Results and Discussion

**Scheme 1:**
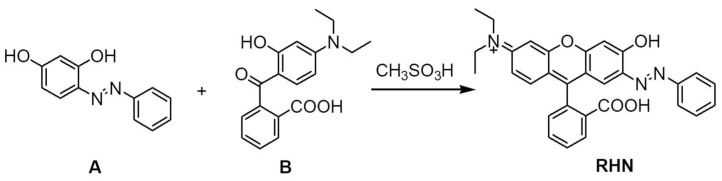
Synthesis of RHN. Reagents and conditions: (E)-4-(2-phenyldiazenyl) benzene-1,3-diol (A), 2-(4-Diethylamino-2-hydroxybenzoyl) benzoic acid (B), CH_3_SO_3_H, 80 °C, overnight.

The designed probe RHN was synthesized by a one-step chemical synthesis route, described in Scheme 1. Both 2-(4-Diethylamino-2-hydroxybenzoyl)benzoic acid and (E)-4-(2-phenyldiazenyl)benzene-1,3-diol underwent condensation and rearrangement reactions in methanesulfonic acid to obtain the target product RHN in higher yield. The NMR and HRMS were performed to verify and determine the structure of RHN, and the data are presented in the Supplementary Materials.

**Figure 1:**
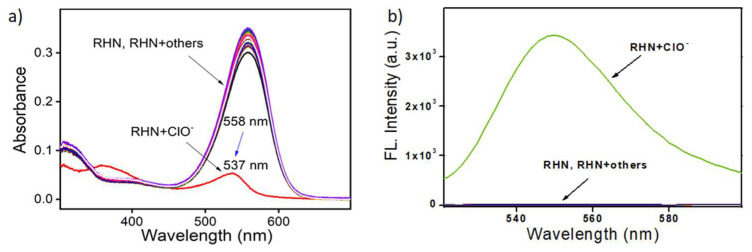
(**a**) Absorption spectra response of RHN (10 μM; 10 mM, pH = 7.4, PBS) upon addition of different species (10 equiv.); (**b**) Emission spectra response of RHN (10 μM; 10 mM, pH = 7.4, PBS) upon addition of different species (10 equiv.). Different species: (1) ^1^O_2_; (2) Cys; (3) GSH; (4) H_2_O_2_; (5) ClO^−^; (6) Hcy; (7) HSO_3_^−^_;_ (8) HSO_4_^−^; (9) TBHP; (10) NO; (11) NO_2_^−^; (12) NO_3_^−^; (13) O_2_^−^; (14) •OH; (15) ONOO^−^; (16) S^2−^; (17) S_2_O_3_^2−^; (18) S_2_O_4_^2−^; (19) S_2_O_8_^2−^; (20) SO_3_^2−^; (21) SO_4_^2−^; (22) blank. The conditions: PBS buffer (10 mM, pH = 7.4); λ_ex_ = 500 nm; slits: 10/5 nm.

**Scheme 2:**
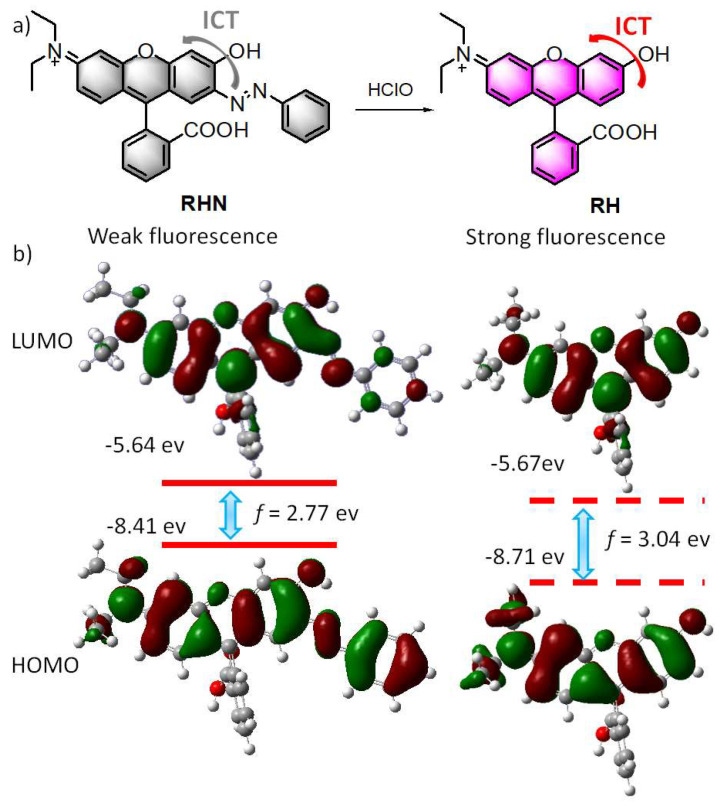
(**a**) The possible reaction mechanism; (**b**) The LUMO and HOMO of RHN and RH calculated with DFT at the B3LYP/6-31G* level using Gaussian 09.

After obtaining RHN, the ability of RHN to recognize HClO was assessed. At the beginning, the selectivity of RHN to HClO has been explored in the presence of different oxide species (^1^O_2_, H_2_O_2_, ClO^−^, TBHP, NO, NO_2_^−^, NO_3_^−^, O_2_^−^, •OH, ONOO^−^) and sulfur-containing compounds (Cys, GSH, Hcy, HSO_3_^−^_,_ HSO_4_^−^, S^2−^, S_2_O_3_^2−^, S_2_O_4_^2−^, S_2_O_8_^2−^, SO_3_^2−^, SO_4_^2−^). As shown in Figure 1a, upon addition of ClO^−^, the maximum absorption band of RHN was blue-shifted from 558 nm to 537 nm, and the maximum absorption intensity was significantly decreased. In contrast, upon addition of other above-mentioned substances, the absorption intensity of RHN fluctuated significantly less than those of the addition of HClO. Meanwhile, the maximum emission intensity at 550 nm was significantly enhanced after the addition of ClO^−^. However, the emission intensity at 550 nm basically remained at weak fluorescence after the addition of the other above-mentioned substances. The above experiments implied that RHN has a specific recognition ability for HClO under physiological conditions. In addition, we speculated on the mechanism of the spectral changes. Combined with literature reports [28], the strong electron-withdrawing group phenylazo group of RHN was oxidized and dissociated by HClO to generate Rhodol fluorophore, resulting in inhibited ICT recovery and the fluorescence emission recovery (Scheme 2a). Thus, the DFT calculation at the B3LYP/6-31G* level using Gaussian 09 was carried out, and the LUMO and HOMO of RHN and RH are displayed in Scheme 2b. Compared to the electron clouds in the LUMO of RHN, those of HOMO located over the whole molecular, suggesting an ICT process. Additionally, the energy gaps (*f*) between HOMO and LUMO shifted from 2.77 eV for RHN to 3.04 eV for RH. The DFT results implied that RHN recognizes HClO, based on the ICT mechanism. Furthermore, the HRMS was performed to verify the mechanism. As shown in Figure S1, upon addition of HClO, a new peak emerged at *m*/*z* = 388.1365, which belonged to the RH [C_24_H_22_NO_4_^+^].

**Figure 2:**
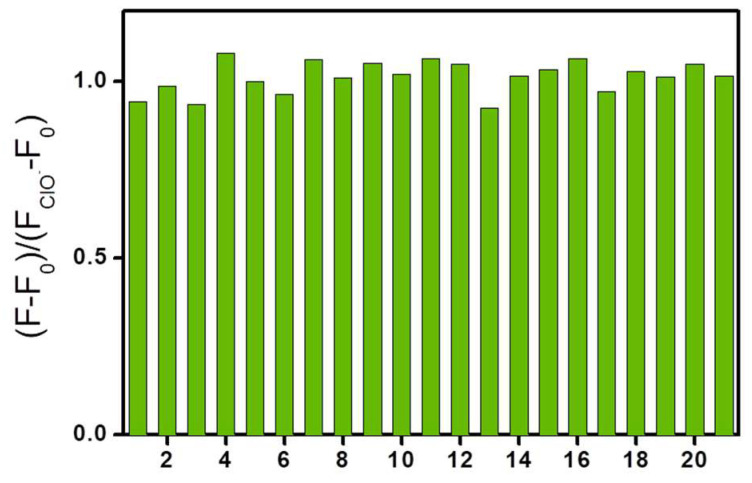
Emission spectra response of RHN (10 μM; 10 mM, pH = 7.4, PBS) upon addition of different species (100 μM) in the presence of ClO^−^ (50 μM). (1) ^1^O_2_; (2) Cys; (3) GSH; (4) H_2_O_2_; (5) blank; (6) Hcy; (7) HSO_3_^−^; (8) HSO_4_^−^; (9) TBHP; (10) NO; (11) NO_2_^−^; (12) NO_3_^−^; (13) O_2_^−^; (14) •OH; (15) ONOO^−^; (16) S^2^^−^; (17) S_2_O_3_^2^^−^; (18) S_2_O_4_^2^^−^; (19) S_2_O_8_^2^^−^; (20) SO_3_^2^^−^; (21) SO_4_^2^^−^. The conditions: PBS buffer (10 mM, pH = 7.4); λ_ex_ = 500 nm; slits: 10/5 nm.

Immediately, competition experiments were performed to further explore the specificity of RHN for HClO. As shown in Figure 2, no matter whether it was in the presence of interfering reactive oxygen species or sulfur-containing compounds, RHN still showed an obvious fluorescence response to HClO at 550 nm, which was basically unaffected by these interfering substances. Combining the results of the selectivity and competition experiments, it can be concluded that RHN has high selectivity and can be used for selective recognition of HClO in complex environments, without interference from other substances. 

**Figure 3:**
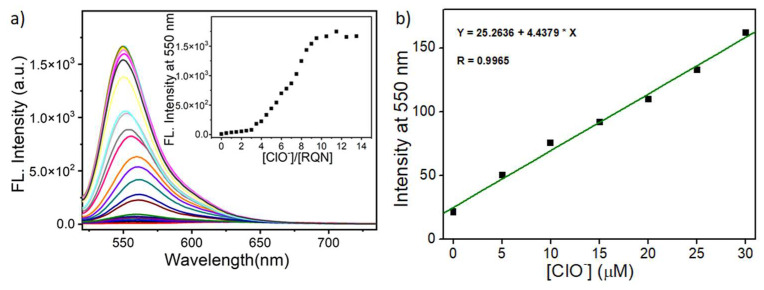
(**a**) Emission spectra response of RHN (10 μM; 10 mM, pH = 7.4, PBS) upon addition of different concentrations of ClO^−^ (0–140 μM); Inset: The plot of I_550_ versus the equivalents of ClO^−^; (**b**) The plot of I_550_ versus the concentrations of ClO^−^ (0–30 μM). The conditions: PBS buffer (10 mM, pH = 7.4); λ_ex_ = 500 nm; slits: 10/5 nm. The emission spectra were collected 10 min after the addition of ClO^−^.

Next, we explored the sensitivity of RHN to recognize HClO by continuous titration experiments. As shown in Figure 3a, with the continuous addition of HClO to the solution system of RHN, the fluorescence emission spectrum of RHN was continuously enhanced, and when the concentration of HClO was 100 μM, the fluorescence emission wavelength of RHN reached the maximum value. Compared with the initial fluorescence emission intensity, the fluorescence emission intensity of RHN at 550 nm increased by about 168 times, and the influence of background emission on the recognition of HClO by RHN was basically negligible. At the same time, the enhancement of fluorescence emission was accompanied by a blueshift of 11 nm. We noticed that the fluorescence emission intensity of RHN increased linearly within 30 μM of the hypochlorous acid concentration. Therefore, we used the fluorescence emission intensity of RHN at 550 nm as the ordinate, and the HClO concentration as the abscissa to the plot. The fitting results in Figure 3b showed that the fluorescence emission intensity of RHN at 550 nm has a good linear relationship with the concentration of HClO, and the fitting constant can reach 0.9965. According to the fitting equation, we could easily obtain the slope. Based on the 3σ/k formula, the detection limit of RHN for HClO was 22 nM, where σ was the standard deviation of 10 consecutive parallel measurements of the fluorescence emission of blank solution of RHN. Compared with the detection limits reported in other literature, the detection limit of RHN was at a low level (Table S1). In addition, the quantum yield of RHN was measured. Upon addition of HClO, the quantum yield of RHN changed from 0.15% to 34%. The results of continuous titration experiments showed that RHN has high sensitivity and can detect trace amounts of HClO in the environment. 

**Figure 4:**
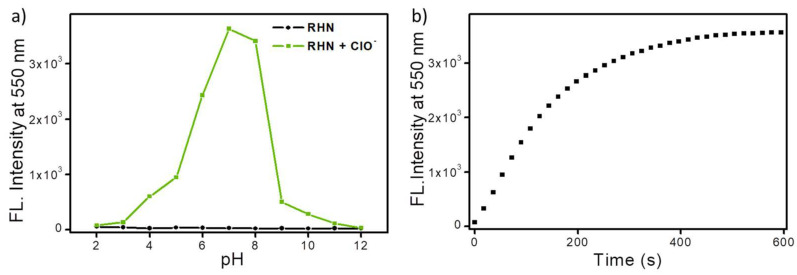
(**a**) Effect of pH on the fluorescence intensity of 10 μM RHN in the absence (black line) and presence of 100 μM ClO^−^ (red line); (**b**) Time dependence of fluorescence intensity of RHN (10 μM) at 550 nm with 10 equiv. of ClO^−^. The conditions: PBS buffer (10 mM, pH = 7.4); λ_ex_ = 500 nm; slits: 10/5 nm.

Finally, in order to evaluate the ability of RHN to monitor HClO in real time in physiological environments, we tested and recorded the ability and time–response curve of RHN to recognize HClO in different pH solutions using a fluorescence spectrophotometer, respectively. As shown in Figure 4a, before the addition of HClO, the fluorescence emission intensity of RHN at 550 nm remained basically unchanged between pH 2–12, indicating that RHN has good chemical stability and will not affect the recognition of HClO. After the addition of HClO, the fluorescence emission intensity of RHN at 550 nm increased significantly between four and nine, indicating that the working pH range of RHN is four to nine, and it has the ability to detect HClO under physiological conditions. In addition, as shown in Figure 4b, when HClO was added to the RHN solution system, the fluorescence emission intensity of RHN at 550 nm increased rapidly and reached a maximum value at about 400 s, indicating that RHN had the ability to monitor HClO in real time. Combined with the above experiments, we can conclude that RHN has potential application capabilities for the real-time detection of HClO under complex physiological conditions. 

**Figure 5:**
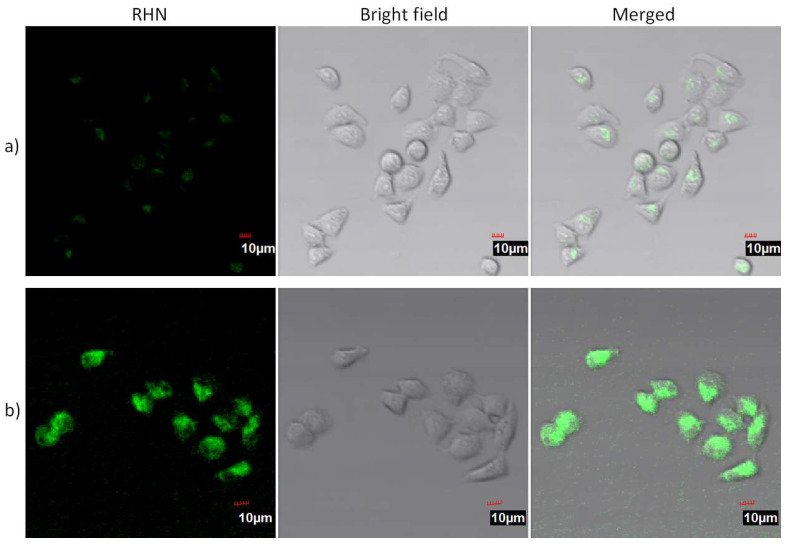
Fluorescence images of HeLa cells. (**a**) Cells were incubated with RHN (1 μM) for 30 min, then (**b**) incubated with OCl^−^ (10 μM) for 30 min. λ_ex_ = 488 nm; λ_em_ = 510–600 nm.

After verifying that RHN has good selectivity, sensitivity and real-time detection capability by spectroscopic experiments, we further explored the application of RHN in cell imaging. The first thing to do was the cytotoxicity assessment. Validation was performed using standard MTT assays. The experimental results showed that, after 24 h of co-culture of different concentrations (0–20 μM) of RHN and cells, the survival rate of HeLa cells could still maintain more than 80% (Figure S2), indicating that RHN has low cytotoxicity. Then, imaging experiments were performed using RHN to stain HeLa cells. In the control group, after co-incubating HeLa cells with RHN (1 μM) in the incubator for 30 min, the culture medium was aspirated and washed three times with PBS to remove excess probe that did not enter the cells, and then imaging experiments were performed under a confocal microscope. Excitation was performed using a 488 nm laser, and the signals were collected in the emission wavelength range between 510–600 nm. The results of the cell imaging experiments showed that RHN showed a weaker signal under the green channel (Figure 5a). In the treated group, RHN (1 μM) was pre-incubated with HeLa cells for half an hour, and the culture medium was aspirated and washed three times with PBS to remove excess probe that did not enter the cells. Hypochlorous acid (10 μM) was then added to the medium for co-culture for half an hour, and the cell imaging experiments were performed under the same conditions. In contrast, this group showed a strong fluorescent signal under the green channel (Figure 5b). The results of the cell imaging experiments showed that RHN has good membrane permeability and can be used to detect HClO in living cells.

**Figure 6:**
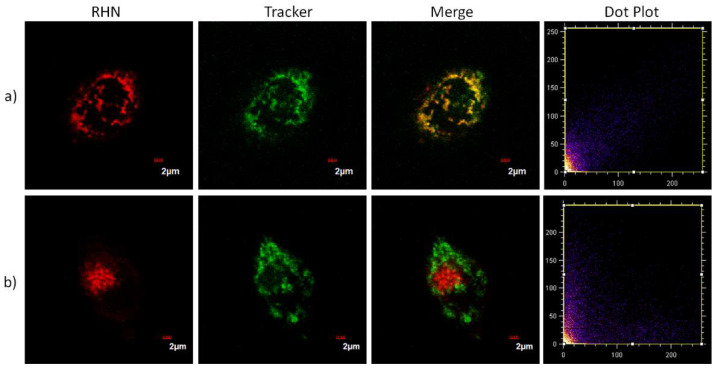
Imaging of HeLa cells costained with RHN (1 μM) upon treatment with MitoTracker^®^ Deep Red FM (200 nM) and LysoTracker^®^ Deep Red (200 nM). Cells were incubated with probes for 30 min, and then treated with OCl^−^ (10 μM) for another 30 min. (**a**) Costained with MitoTracker^®^ Deep Red FM; (**b**) Costained with LysoTracker^®^ Deep Red. Green channel: λ_ex_ = 488 nm; λ_em_ = 510–600 nm; Red channel: λ_ex_ = 559 nm; λ_em_ = 647–671 nm.

Careful observation of the superimposed image of the green channel and the bright field showed that RHN does not evenly fill the entire cytoplasm in the cell, but aggregates at a certain place. According to literature reports, HClO mainly accumulates in mitochondria in cells [5,6], and combined with cation-containing dyes with mitochondrial targeting [29], we judged that RHN is likely to have mitochondrial targeting. To verify the correctness of this judgment, we used commercially available localizers MitoTracker^®^ Deep Red FM (Mito) and LysoTracker^®^ Deep Red (Lyso) to perform colocalization experiments with RHN (1 μM), respectively. After co-incubating RHN and the localization reagents for half an hour, the medium was aspirated and washed three times with PBS buffer to remove excess probes and locating agents. Hypochlorous acid (10 μM) was added and the incubation was continued for half an hour, and imaging experiments were performed under the same conditions using a laser confocal microscope. A 488 nm laser was used for excitation, and the green fluorescence signal of RHN was collected in the wavelength range of 510–600 nm. A 559 nm laser was used for excitation and the red fluorescent signal of the localization reagent emitting in the wavelength range 618–718 nm was collected. As shown in Figure 6a, a large yellow patch could be clearly seen from the superimposed image, indicating that the fluorescence signal of RHN completely overlaps with that of Mito, and they are both distributed in the same region. Dot plots tended to cluster more at the center line. In particular, a high Pearson’s colocalization coefficient (0.89) between them was calculated, proving that both of them are distributed in the same region. In contrast, the fluorescence signal of RHN and the signal of Lyso were clearly distributed in different regions (Figure 6b), as evidenced by the lower Pearson’s colocalization coefficient (0.38). Dot plots tended to show more off-center lines. Colocalization experiments confirmed that RHN could image hypochlorous acid in the mitochondria of living cells and had the ability to detect HClO in mitochondria.

## 3. Materials and Methods

### 3.1. Synthesis

Both 2-(4-Diethylamino-2-hydroxybenzoyl) benzoic acid (688.6 mg, 2.2 mmol) and (*E*)-4-(2-phenyldiazenyl) benzene-1,3-diol (428 mg, 2 mmol) were added to methanesulfonic acid (6 mL) and stirred at 80 °C overnight. After cooling to room temperature, it was neutralized with saturated sodium bicarbonate solution until no bubbles were formed. The mixture was then extracted with dichloromethane (50 mL × 3), and the collected organic layers were dried over anhydrous sodium sulfate. The crude product obtained by vacuum concentration was purified by column chromatography to finally obtain pure solid compound RHN (777.3 mg, 79%). The NMR was performed to verify and determine the structure of RHN; ^1^H NMR (400 MHz, CDCl_3_) δ 13.25 (s, 1H), 8.07 (d, J = 7.4 Hz, 1H), 7.75 (d, J = 6.7 Hz, 2H), 7.71–7.63 (m, 2H), 7.45 (t, J = 7.3 Hz, 3H), 7.39 (s, 1H), 7.28 (s, 1H), 6.85 (s, 1H), 6.60 (d, J = 8.8 Hz, 1H), 6.49 (s, 1H), 6.39 (d, J = 7.2 Hz, 1H), 3.37 (dd, J = 13.8, 6.8 Hz, 4H), 1.19 (t, J = 7.0 Hz, 6H); ^13^C NMR (100 MHz, CDCl_3_) δ 169.53, 155.30, 154.94, 152.77, 152.62, 150.26, 135.04, 134.70, 134.45, 131.15, 129.77, 129.39, 129.04, 128.97, 127.29, 125.13, 124.08, 122.12, 112.91, 104.86, 65.60, 44.56, 12.48. HRMS: (C_30_H_26_N_3_O_4_^+^) *m*/*z*: calculated for [M]^+^: 492.1923. Found [M]^+^: 492.1920. 

### 3.2. Standard MTT Assay

The MTT assay was performed, according to our previous methods [28,29]. HeLa cells were inoculated and allowed to adhere for 12 h. Then, RHN (0, 1, 3, 5, 10 and 20 μM) were added to the cells and cultured for another 24 h. The solution was changed and washed three times with PBS, and then 3-(4,5-dimethylthiazol-2-yl)-2,5-diphenyltetrazolium bromide was added and cultured for another 4 h. The culture solution was changed again and washed before adding DMSO, and then the absorbencies were measured at 490 nm after standing for 2 h. 

### 3.3. Cell Culture and Fluorescence Imaging

HeLa cells were cultured, according to our previous methods [30,31]. The cells were incubated with RHN (1 μM) for 30 min, and then ClO^−^ (10 μM) was added and incubated for another 30 min. For co-localization experiments, RHN (1 μM) were incubated with MitoTracker^®^ Deep Red FM (200 nM) and LysoTracker^®^ Deep Red (200 nM) for 30 min, respectively, and then treated with ClO^−^ (10 μM) for another 30 min. 

## 4. Conclusions

In conclusion, we rationally designed and synthesized a novel phenylazo-based fluorescent probe for the sensing and imaging of hypochlorous acid in mitochondria in living cells. As expected, RHN exhibited high selectivity, high sensitivity and rapid response with detection limits as low as 22 nM. Importantly, the results of the cell imaging experiments indicated that RHN has the ability to image and sense HClO in mitochondria, which is of great significance for the exploration specific role of HClO in the immune system and diseases. 

## Data Availability

Not applicable.

## Supplementary Materials

The following supporting information can be downloaded at: https://www.mdpi.com/article/10.3390/molecules27092978/s1, Figure S1. The HRMS of RHN after addition of HClO; Figure S2: MTT assay for the survival rate of HeLa cells treated with various concentrations of RHN for 24 h; Figures S3–S5: ^1^H, ^13^C NMR spectra and HRMS of RHN; Table S1: The previous results of other HClO probes. Table S2: Compared with RHN, the data of other HClO probes.
