# Supplementary material for: A New Phenylazo-Based Fluorescent Probe for Sensitive Detection of Hypochlorous Acid in Aqueous Solution
Source: Molecules. 2022 May 6;27(9):2978. doi: 10.3390/molecules27092978 (PMC9102862; doi:10.3390/molecules27092978)
Supplement: Supplementary file 1 [file molecules-27-02978-s001.zip › molecules-1650581-supplementary.pdf]

molecules

Supporting Information

A New Phenylazo-Based Fluorescent Probe for Sensitive Detection of Hypochlorous Acid in Aqueous Solution

Qiuchen Liu [1,2,3], Chang Liu [2], Song He [2], Liancheng Zhao [2,4], Xianshun Zeng [2,4,*], Jin Zhou [1,*] and Jin Gong [1,2,*]

[1] School of Pharmacy, Weifang Medical University, Weifang 261053, China; qiuchen-liu@163.com
[2] Tianjin Key Laboratory for Photoelectric Materials and Devices, School of Materials Science & Engineering, Tianjin University of Technology, Tianjin 300384, China; kmn667@163.com (C.L.); hesong@tjut.edu.cn (S.H.); lczhao@hit.edu.cn (L.Z.)
[3] School of Chemical Engineering and Technology, Tianjin University, Tianjin 300072, China
[4] School of Materials Science and Engineering, Harbin Institute of Technology, Harbin 150001, China
* Correspondence: xshzeng@tjut.edu.cn (X.Z.); zhoujin@wfmc.edu.cn (J.Z.); gongjin@wfmc.edu.cn (J.G.)

Contents

Table S1. The previous results of other HClO probes..S2–S3
Table S2. The data of other HClO probes..S3–S4
The HRMS of RHN after addition of HClO..S4
MTT assay...S5
^{1}H, ^{13}C NMR spectra and HRMS of RHN...S5–S6

Table S1. The previous results of other HClO probes.

No.	Structures	Solution	F/F_0	LOD (*nM*)	Time (*s*)	*Ref.*
1	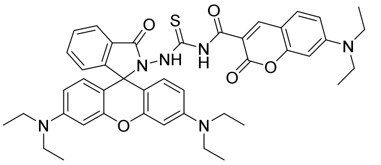	NaH_2PO_4:EtOH (2: 8, v/v)	2	660	60	*Sens. Actuators B Chem., 2017, 247, 736*
2	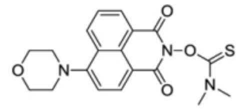	DMSO:PBS (1:99, v/v)	9	105.2	16	*J. Photochem. Photobiol. A: Chem. 2020, 392, 112399*
3	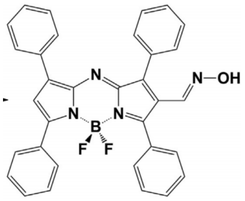	HEPES:CH_3CN (1:1, v/v)	6	2330	--	*Spectrochim. Acta A Mol. Biomol. Spectrosc., 2019, 206, 190*
4	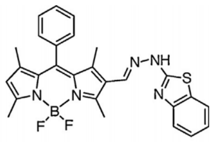	PBS:MeOH (1:1, v/v)	41	2.4	--	*Anal. Chim. Acta 2015, 882, 68*
5	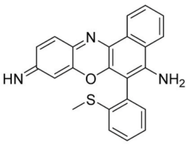	PBS	16	94.7	Seconds	*Talanta 2021, 226, 122152*
6	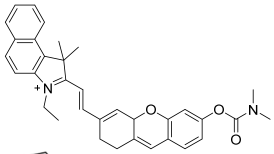	DMSO:PBS (1:9, v/v)	15	35	30	*Dyes Pigm., 2021, 187, 109145.*
7	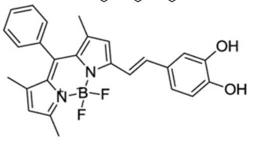	Acetonitrile: PBS (1:9, v/v)	15	4.3	900	*Chem. Commun., 2018, 54, 1849*
8	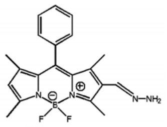	PBS:EtOH (1:1, v/v)	9	56	1800	*RSC Adv., 2015, 5, 73040*
9	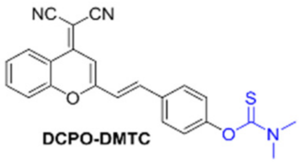	DMSO:PBS (1:1, v/v)	15	164	300	*Talanta, 2019, 196, 352*
10	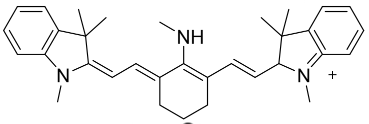	PBS	20	100	--	*Chem. Sci., 2018, 9, 8207*
11	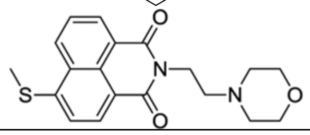	PBS	20	674	150	*Anal. Chem., 2017, 89, 10384*

**12**	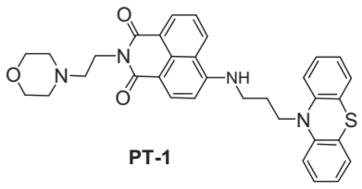	H_2O:EtOH (1:1, v/v)	160	10^{-10} M	10	*Talanta, 2017, 174, 234*

Table S2. Compared with RHN, the data of other HClO probes.

No.	Structures	Solution	LOD (*nM*)	Time (*s*)	*Ref.*
**1**	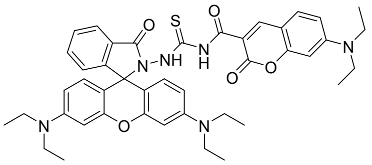	NaH_2PO_4:EtOH (2: 8, v/v)	660	60	*Sens. Actuators B Chem., 2017, 247, 736*
**2**	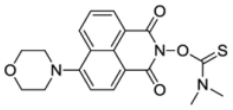	DMSO:PBS (1:99, v/v)	105.2	16	*J. Photochem. Photobiol. A: Chem. 2020, 392, 112399*
**3**	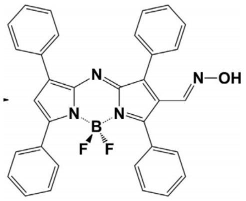	HEPES:CH_3CN (1:1, v/v)	2330	--	*Spectrochim. Acta A Mol. Biomol. Spectrosc., 2019, 206, 190*
**4**	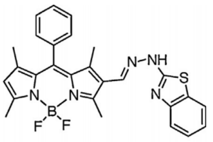	PBS:MeOH (1:1, v/v)	2.4	--	*Anal. Chim. Acta 2015, 882, 68*
**5**	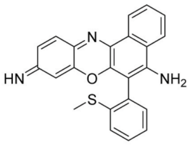	PBS	94.7	Seconds	*Talanta 2021, 226, 122152*
**6**	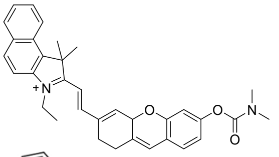	DMSO:PBS (1:9, v/v)	35	30	*Dyes Pigm., 2021, 187, 109145.*
**7**	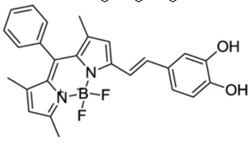	Acetonitrile: PBS (1:9, v/v)	4.3	900	*Chem. Commun., 2018, 54, 1849*
**8**	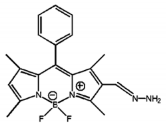	PBS:EtOH (1:1, v/v)	56	1800	*RSC Adv., 2015, 5, 73040*
**9**	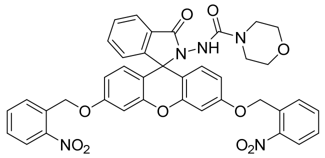	PBS:DMF (4:1, v/v)	62	--	*Chem. Commun., 2018, 54, 9238*
**10**	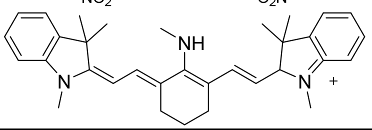	PBS	100	--	*Chem. Sci., 2018, 9, 8207*

**11**	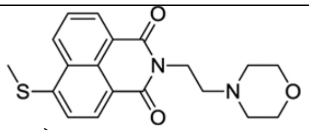	PBS	674	150	*Anal. Chem., 2017, 89, 10384*
**12**	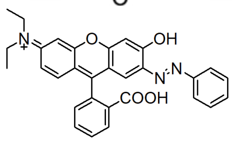 RHN	**PBS**	**22**	**400**	***This work***

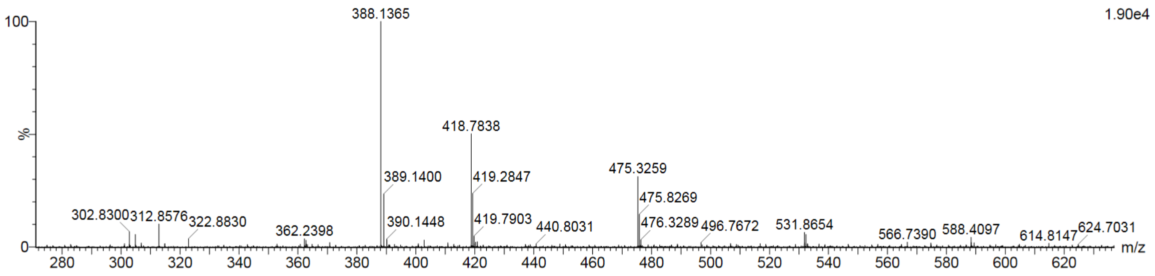

Figure S1. The HRMS of RHN after addition of HClO.

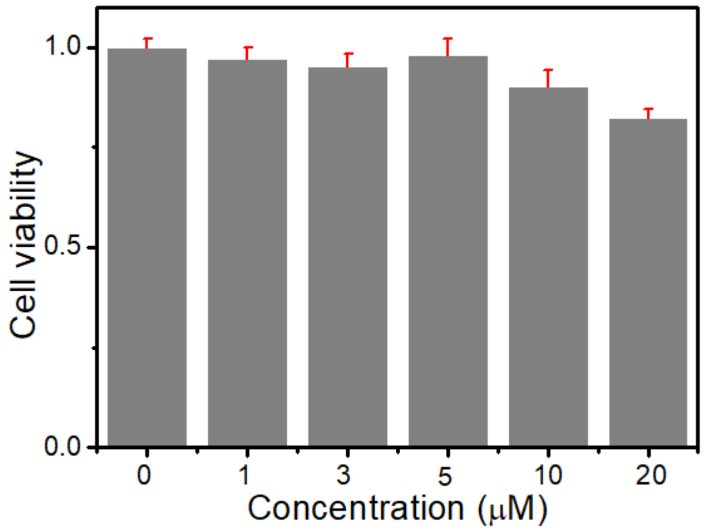

Figure S2. MTT assay for the survival rate of HeLa cells treated with various concentrations of RHN for 24 h. Error bars represent the standard deviations of 5 trials.

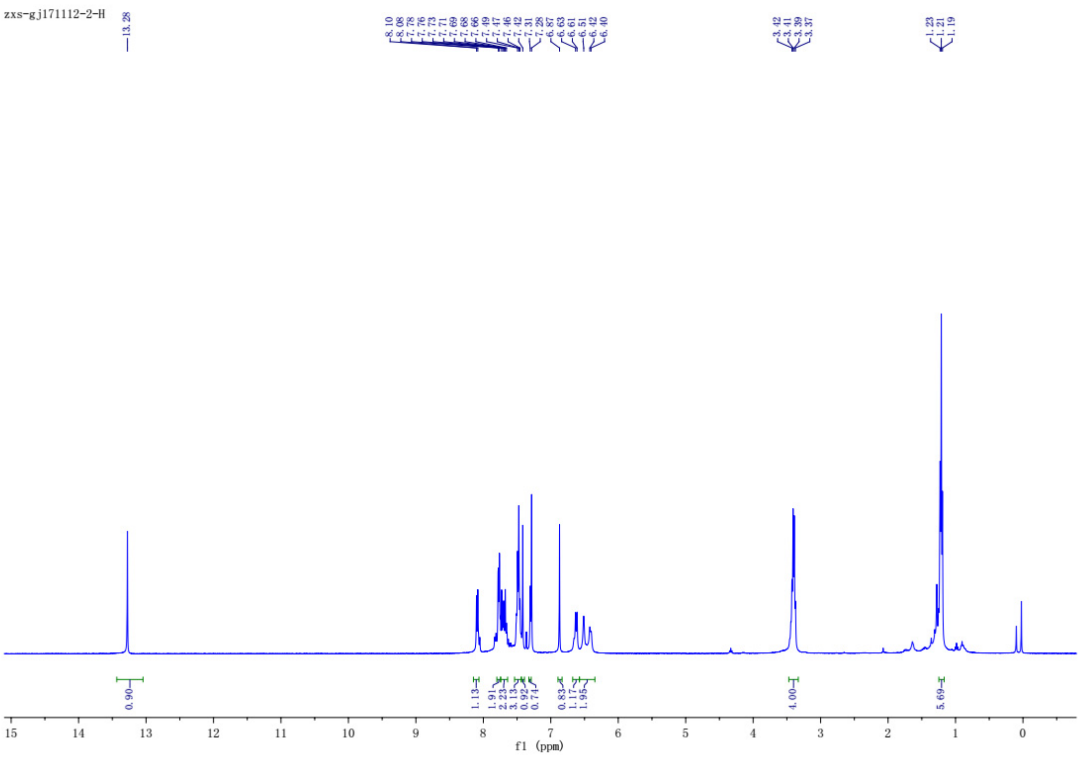

Figure S3. ^{1}H NMR spectra of compound RHN in $CDCl_3$.

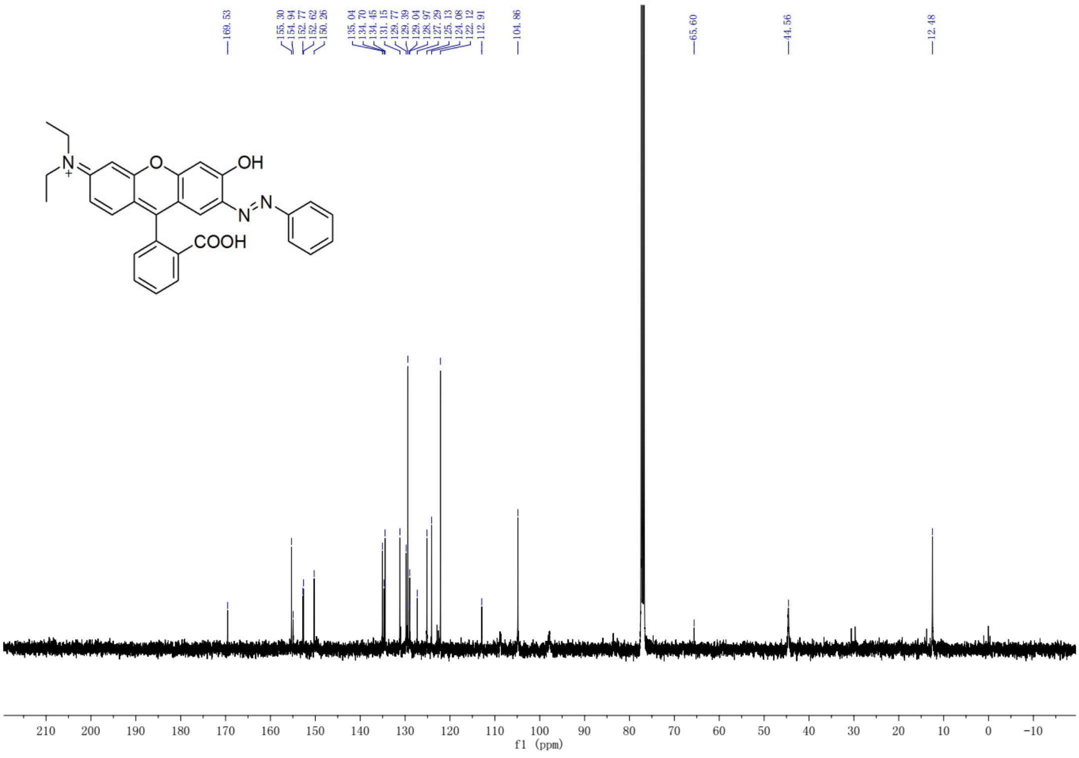

Figure S4. ^{13}C NMR spectra of compound RHN in $CDCl_3$.

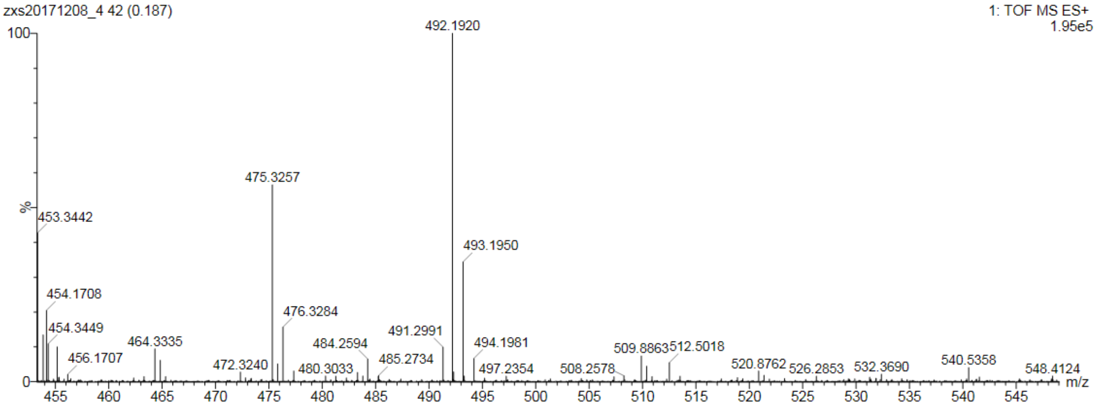

Figure S5. HRMS spectrum of compound RHN.
